# A Label-Free Immunosensor for Ultrasensitive Detection of Ketamine Based on Quartz Crystal Microbalance

**DOI:** 10.3390/s150408540

**Published:** 2015-04-13

**Authors:** Ya Yang, Yifeng Tu, Xiaoshu Wang, Jinyin Pan, Yun Ding

**Affiliations:** 1 Institute of Analytical Chemistry, Soochow University, Dushu Lake Campus of Soochow University, Suzhou Industrial Park, Suzhou 215123, China; E-Mail: fenghuayunji@163.com; 2 Department of Forensic Medicine, Soochow University, Dushu Lake Campus of Soochow University, Suzhou Industrial Park, Suzhou 215123, China; E-Mails: wangxiaoshu@suda.edu.cn (X.W.); jiangnaiyunzi@sina.com (Y.D.); 3 The Key Lab of Health Chemistry and Molecular Diagnosis of Suzhou, Dushu Lake Campus of Soochow University, Suzhou Industrial Park, Suzhou 215123, China

**Keywords:** quartz crystal microbalance, Ketamine, immunosensor, label-free, on-spot detection

## Abstract

In this study, we have developed a label-free immunosensor with the variation of resonance frequency (Δf) of a quartz crystal microbalance (QCM) as readout signal for ultrasensitive detection of Ketamine (KT). An optimized strategy for immobilization of KT antibody on the surface of the QCM chip was implemented via the self-assembly modification of 3-mercaptopropionic acid, and then activated with 1-ethyl-3- (3-dimethylaminoprophl) carbodiimide and n-hydroxysuccinimide. The specific affinity between the antibody and the antigen ensured a selective response toward KT. The Δf linearly related to the concentration of KT in the range of 1 to 40 pg/mL, with a detection limit of 0.86 pg/mL (S/N = 3). The obtained immunosensor was applied to detect the KT in spiked human urine without any pretreatment but dilution with recoveries from 91.8% to 108%. The developed sensor is promising to perform the portable or on-spot KT detection in clinic or forensic cases.

## 1. Introduction

Ketamine ((*RS*)-2-(2-chlorophenyl)-2-(methylamino)cyclohexanone, KT), an NMDA receptor antagonist first developed in 1962, was once used as an anesthetic agent in veterinary and human surgery in 1970s because of its few side effects and short actuation duration [1,2,3]. But, in the last 20 years, as a recreational drug with its hallucinogenic and dissociative effects, it has been abused all over the world [4] and thereafter controlled in many countries. The high-dose intake of KT will result in similar effects to that of phencyclidine or lysergide including harming the central nervous system [5]. Because of this serious situation, its detection is very important in clinic or forensic field. To-date, many analytical strategies for KT detection have been reported, such as enzyme-linked immunosorbent assay (ELISA) [6,7], gas chromatography-mass spectrometry (GC-MS) [8,9], high performance liquid chromatography (HPLC) [10], liquid chromatography-mass spectrometry-mass spectrometry (LC-MS-MS) [11,12,13], headspace-solid phase microextraction-gas chromatography-mass spectrometry (HS-SPME-GC-MS) [14,15] and electrochemical methods [16,17,18]. Herein, the ELISA method is sensitive and most suitable for routine examination of KT in clinical or forensic cases. However, most of these methods require sophisticated and heavy instruments with time-consuming procedure, which makes them not suitable for portable or on-spot examination. Devices with rapid, selective, sensitive response, particularly for the fast screening in point-of-care test of KT are in demand.

Biosensors have been widely applied in chemical, clinical and environmental analysis because of their rapid, sensitive and selective responses [19,20,21]. Electrochemical immunosensor is one example of biosensor which has the advantages including simple structure, easy to use, feasible miniaturization and the possibility of low-cost mass-production [22,23]. In order to achieve a low detection limit, a lot of efforts have been devoted to improve the signal-to-noise ratio of the electrochemical output [24,25]. Thereinto quartz crystal microbalance (QCM), a gravimetric sensing technique based on piezoelectric effect, is also a promising signaling protocol for biosensor. The regression of the variation of resonant frequency (Δf) upon the surface mass change (Δm) of QCM chip has been affirmed by Sauerbrey in 1959 [26]. Sensitive response can be obtained because of the high quality factor of quartz crystal (10^5^–10^6^). In recent years, it has been intensively reported for a lot of applications, such as monitoring of environmental contaminants, biosensor developing and drug analysis *etc.* [27,28,29]. It is also suitable for signaling the antibody-antigen interaction.

In this study, a KT immunosensor with QCM response is developed. It was constructed by immobilizing the KT antibody onto the surface of a QCM chip [30,31]. First a self-assembly monolayer (SAM) of 3-mercaptopropionic acid (3-MPA) was formed on a QCM chip. By activation of the SAM layer via the reaction involving 3-(3-dimethylaminopropl)-1-ethylcarbodiimide hydrochloride (EDC) and n-hydroxysuccinimide (NHS), an amide bond was formed between the carboxylic acid group of 3-MPA and the amine group of KT antibody. In this way, the KT antibody was immobilized on the QCM chip [32]. The quantifying character of developed sensor was then verified by detecting the KT in spiked human urine.

## 2. Experimental Section

### 2.1. Reagents and Materials

KT hydrochloride injection was purchased from Jiangsu Hengrui Medicine Co. Ltd (Lianyungang, China). KT monoclonal antibody (1 mg/mL) was from Fankel Co. Ltd (Shanghai, China). 3-mercaptopropionic acid (3-MPA) (>99%) was from Alfa Aesar (Tianjin) Chemicals Co. Ltd. (Beijing, China). NHS (98%) was obtained from Fluka (Buchs, Switzerland). EDC (98%) was purchased from Sigma-Aldrich Co. (St. Louis, MO, USA). All reagents were used as received without further purification. Ultrapure water was used throughout the experiments. Phosphate buffered saline (PBS) (0.01 mol/L, pH 7.4) was used to dilute all solutions.

### 2.2. Apparatus

The QCM measurements were performed on a CHI400A electrochemical workstation (Chenhua Instruments Co. Ltd. Shanghai, China) under acquiescent conditions. The QCM chip is a thin AT-cut quartz wafer coated with Au electrode on each side. The measurements of electrochemical impedance spectroscopy (EIS) were executed on an RST5200 electrochemical workstation (Suzhou Risetest Instrument Co. Ltd., Suzhou, China) with a three-electrode cell.

### 2.3. Fabrication of the Immunosensor

The QCM-chip was cleaned with chromosulfuric acid repeatedly and then flushed with ultrapure water and dried by nitrogen flush. The treated QCM-chip was then immersed in PBS (pH = 7.4) containing 10 mmol/L 3-MPA for 12 h to carry out the self-assembly modification. Excess 3-MPA was removed by rinsing with PBS before being placed in PBS containing EDC (3.2 mmol/L) and NHS (0.4 mmol/L) for a 4 h activation. After rinsing with ultrapure water thoroughly and dried under a nitrogen stream, adequate amounted KT-antibody solution (180 μL of 1:150 diluted solution) was applied on its surface and then kept in a humid environment at 4 °C for 2 h to covalently bind the antibody. Excessive antibody was washed off by PBS. The sensor was then stored at 4 °C.

### 2.4. The Electrochemical Measurements

The electric resistance of QCM-chip changed during the assembly process. In this work, EIS was carried out to characterize and confirm the sensor constructing. The experiments were performed in a 0.1 mol/L NaCl solution containing 5 mmol/L [Fe(CN)_6_]^4−/3−^ after superimposing a 5 mV AC perturbation voltage over the frequency range between 1 Hz and 100 MHz at room temperature. To obtain satisfactory results, all QCM measurements were carried out in a shockproof and electromagnetic-shielding environment.

### 2.5. Detection of Ketamine in Urine Matrix

To evaluate the practicability of the developed KT immunosensor, it was placed in service to detect the KT in a human urine matrix under optimal detection conditions. KT detection was also carried out in the presence of potential interfering species including urea, uric acid and ammonia to verify its specificity. The recovery result of the KT immunosensor was obtained by standard addition measurements. To identify the stability of the immunosensor, by storing at 4 °C, the sensor was repeatedly tested with the interval of every 24 h. The stability is evaluated by the comparison of frequency output with its initial value.

## 3. Results and Discussion

### 3.1. To Verify the Immunosensor Preparation

**Figure 1:**
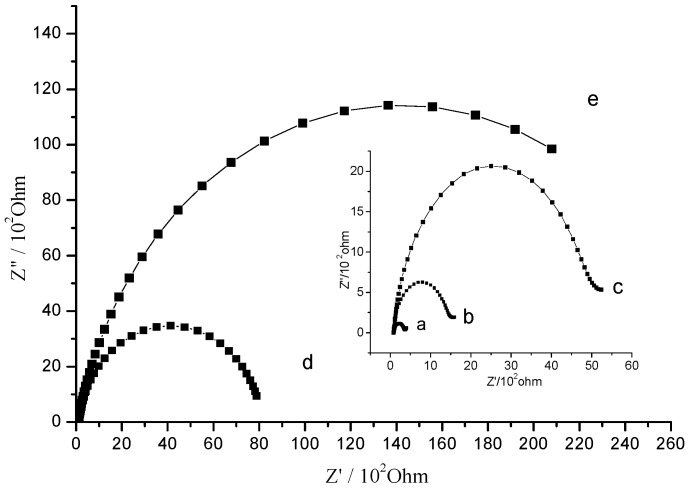
The EIS curves of (**a**) bare QCM chip; (**b**) modified with 3-MPA; (**c**) activated by EDC/NHS and (**d**/**e**) covalently bound with degrees of KT-Ab in 0.1 mol/L NaCl solution containing 5 mmol/L Fe(CN)_6_^4−/3−^. AC perturbation voltage: 5 mV, 1 Hz to 100 MHz.

EIS is effective for probing the properties of the sensing-matrix/solution interface [33]. In this work, we used it to verify the assembling of each component of the immunosensor. After each step of the surface modification, the change of the surface insulation of the quartz chip was investigated with Fe(CN)_6_^4−^/^3−^ as electrochemical probe (Figure 1). In curve “a”, an electron transfer resistance of 116 Ω, estimated by the semicircle diameter, denoted a fast electron transfer. After immobilizing a 3-MPA SAM on the Au chip surface, a kinetic barrier for the electron transfer was encountered, resulted in a larger electron transfer resistance of 1353 Ω, as shown in curve “b”. During the EDC/NHS activation step [17], the carboxylic group of the SAM were replaced by amino group from EDC and then resulted in an electrostatic interaction with the negatively charged hydroxyl terminal of NHS with a resistance of 3652 Ω, as shown in curve “c”. This substrate was then ready for immobilization of the KT antibody [34]. Once the antibody was immobilized on its surface, the resistance greatly increased (curve “d”, 8070 Ω) owing to the inhibition of electron transfer by those giant biological molecules. The quantity of immobilized antibody affected the performance of the immunosensor. When all activated sites were saturated, the resistance got its greatest value (28,270 Ω) and the immunosensor was full functionalized, as shown in curve “e”.

### 3.2. Effects of Immobilized KT-Ab Quantity on Sensing Performance

**Figure 2:**
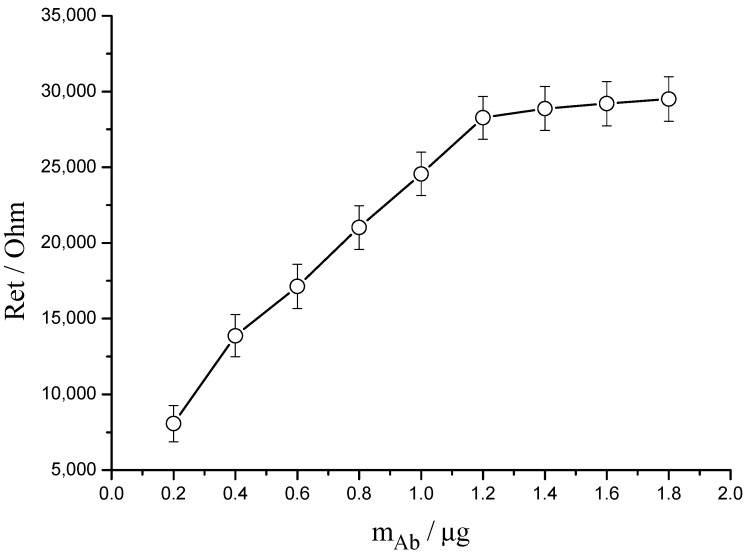
The effect of immobilized KT antibody quantity on sensing performance. The EIS values were obtained in 0.1 mol/L NaCl solution containing 5 mmol/L Fe(CN)_6_^4−/3−^, with 5 mV of AC perturbation voltage, 1 Hz to 100 MHz.

The quantity of KT antibody bound on the surface of quartz chip was an important factor which affected the performance of resultant immunosensor. Under the optimized experimental conditions including the time of 3-MPA self-assembly, the time of EDC/NHS activation and the ratio of EDC/NHS which were discussed in our previous study [17], the effect of immobilized KT antibody quantity on sensing performance was investigated. As Figure 2 shown, with the deposition of larger quantity of antibody onto the surface of quartz chip, the resistance increased until a plateau was obtained, which meant enough antibodies completely occupying activated sites.

### 3.3. Responses of the Developed Immunosensor toward KT

**Figure 3:**
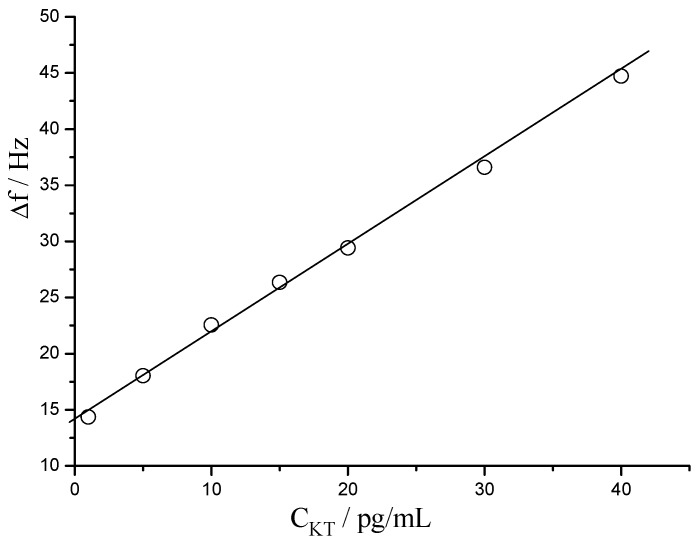
The calibration of response of resonant frequency toward KT concentration.

As a typical label-free technique, the resultant sensor was directly incubated in every differently concentrated KT solutions for immuno-interaction with the period of 10 min, then rinsed with PBS and dried by a nitrogen stream. After that, the QCM output was recorded. Within the concentration range of KT from 1 to 40 pg/mL, the regression equation relating the change of resonant frequency (Δf) and KT concentration (C_KT_) is Δf = 14.3 + 0.76C_KT_ (*r* = 0.998), with a detection limit (LOD) of 0.86 pg/mL (S/N = 3), as shown in Figure 3. The Table 1 lists the relative standard deviation (RSD) associated to each concentration of KT (*n* = 5), exhibits quite advisable level.

As compared with other reported methods for the detection of KT, the resultant immunosensor obtained here showed remarkably higher sensitivity besides one work of ourselves [17], as shown in Table 2. In our work, we have found that no matter the technique of QCM or EIS both had high sensitivity transcend the reported papers which would enable the real application. However, for current QCM technique, despite the narrow gap on sensitivity compared to EIS, it is simpler and faster for use.

**Table 1:** The relative standard deviation associated to each concentration.

Concentration (pg/mL)	1	5	10	15	20	30	40
RSD (%)	8.83	5.12	2.32	2.17	2.45	4.47	4.74

**Table 2:** The comparison of different analytical methods for the detection of KT.

Detection Method	LOD	Sample	The Linear Range	Reference
GC/MS	10 ng/mL	Urine	30–1000 ng/mL	[8]
LC-MS-MS	5 ng/mL	Urine	5–1000 ng/mL	[12]
electrochemistry	5 ng/mL	Beverage	50–2000 ng/mL	[16]
EIS	0.1 pg/mL	Serum	1–100 pmol/L	[17]
QCM	0.86pg/mL	Urine	1–40 pg/mL	Present work

### 3.4. The Anti-Interference Ability and Stability of the Resulted Immunosensor

**Figure 4:**
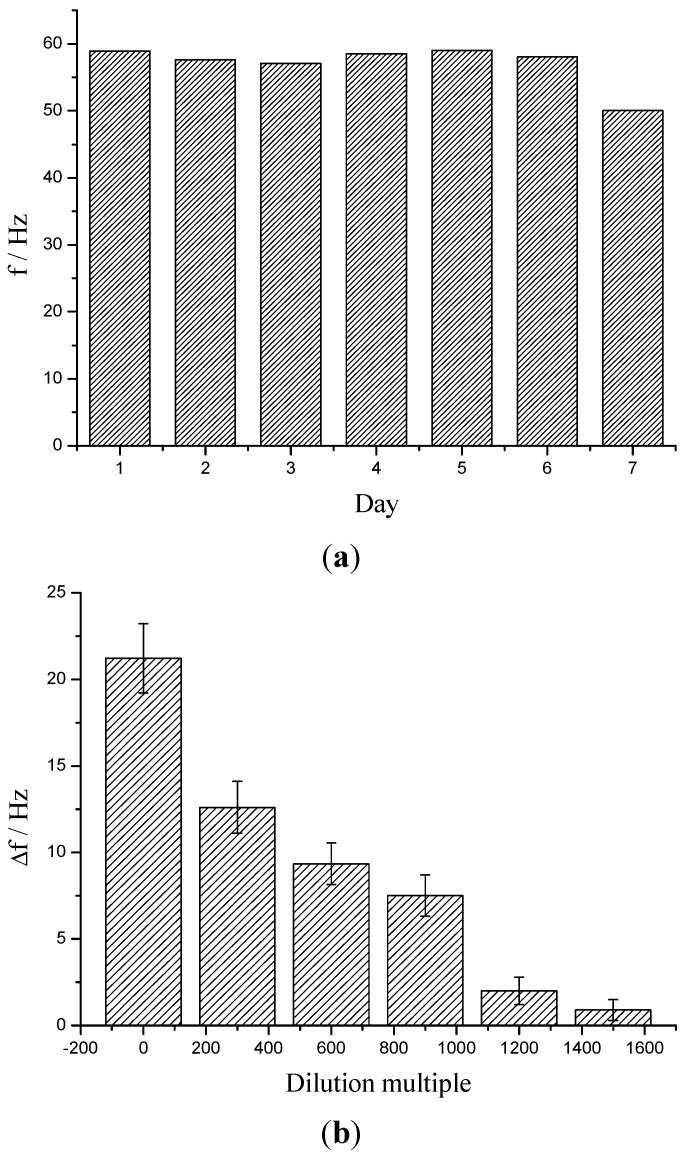
(**a**) The frequency output of resultant KT immunosensor during 7 days; (**b**) the interference of human urine matrix along with the dilution multiple.

The stability of the resulted immunosensor was investigated by comparing its resonant frequency output along with the time of use. In 6 days, if stored at 4 °C after each test, there was no significant change to its initial value (RSD < 2%), as showing in Figure 4a.

The highly concentrated components of urine such as urea, uric acid and ammonia will be the primary potential interferences for KT detection. As shown in Table 3, the resultant sensor had excellent anti-interference ability to those components for at least 25,000 of tolerance. Considering the great disparity of the concentrations of those potential interferents in urine for more than millions multiple, the urine samples were considered to be diluted to avoid their interference and no other pretreatment was required. The results suggested that 1500 multiple was enough (it caused only 4.04% of Δf) (see Figure 4b). Under this situation, the sensor still provided detection down to ng/mL level. This is enough for the analysis of real samples, which normally contained the KT at μg/mL level (relied on oral or injection doping).

**Table 3:** The influence of concentrated urea, uric acid and ammonia on the detection of 20 pg/mL of KT.

Compound	Concentration (ng/mL)	Δf (Hz)	Influence (Relative Error)
urea	50	0.04	0.14%
urea	500	2.49	8.5%
uric acid	50	1.02	3.5%
uric acid	500	2.83	9.6%
ammonia	50	0.32	1.1%
ammonia	500	2.39	8.1%

### 3.5. Determination of KT in Spiked Urine Sample

Urine is the most readily available sample to test for the intake of KT for either clinical or forensic purposes. Urine sample, from a volunteer, spiked with KT at different concentration, was used for the validation of the practicability of the developed sensor. After a 1500-fold dilution, then incubated on sensor, the response of resonant frequency was measured. Satisfactory recoveries were obtained (listed in Table 4), which demonstrated the reliability of the developed KT immunosensor.

**Table 4:** The detected results and recoveries of KT in spiked urine samples.

In Spiked Sample (pg/mL)	After Dilution (pg/mL)	Δf (Hz)	Detected Concentration (pg/mL)	Recovery
3,000	2.0	15.9	2.14	107%
6,000	4.0	17.1	3.67	91.8%
12,000	8.0	20.9	8.66	108%
24,000	16.0	26.1	15.5	96.9%
48,000	32.0	36.7	29.5	92.2%

## 4. Conclusions

In this work, an ultrasensitive QCM immunosensor for the detection of KT was successfully developed. It was prepared by immobilizing KT antibody on the surface of a modified QCM chip. The preparing steps were monitored by EIS. The results indicated that the obtained immunosensor had good stability, great anti-interference ability and the detection limit at the level of pg/mL for KT detection. The developed QCM immunosensor can be applied to label-free analysis of KT in urine samples for forensic or clinical cases.
